# *MRPS18CP2 *alleles and *DEFA3 *absence as putative chromosome 8p23.1 modifiers of hearing loss due to mtDNA mutation A1555G in the 12S rRNA gene

**DOI:** 10.1186/1471-2350-8-81

**Published:** 2007-12-21

**Authors:** Ester Ballana, Josep Maria Mercader, Nathan Fischel-Ghodsian, Xavier Estivill

**Affiliations:** 1 Genes and Disease Program, Centre for Genomic Regulation (CRG), Barcelona, Catalonia, Spain; 2 CIBER en Epidemiología y Salud Pública (CIBERESP), Barcelona, Catalonia, Spain; 3 Cedars-Sinai Medical Center and David Geffen School of Medicine at UCLA, Los Angeles, USA; 4 CeGen, Spanish National Genotyping Centre, Barcelona, Catalonia, Spain; 5 Universitat Pompeu Fabra (UPF), Barcelona, Catalonia, Spain

## Abstract

**Background:**

Mitochondrial DNA (mtDNA) mutations account for at least 5% of cases of postlingual, nonsyndromic hearing impairment. Among them, mutation A1555G is frequently found associated with aminoglycoside-induced and/or nonsyndromic hearing loss in families presenting with extremely variable clinical phenotypes. Biochemical and genetic data have suggested that nuclear background is the main factor involved in modulating the phenotypic expression of mutation A1555G. However, although a major nuclear modifying locus was located on chromosome 8p23.1 and regardless intensive screening of the region, the gene involved has not been identified.

**Methods:**

With the aim to gain insights into the factors that determine the phenotypic expression of A1555G mutation, we have analysed in detail different genetic and genomic elements on 8p23.1 region (*DEFA3 *gene absence, *CLDN23 *gene and *MRPS18CP2 *pseudogene) in a group of 213 A1555G carriers.

**Results:**

Family based association studies identified a positive association for a polymorphism on *MRPS18CP2 *and an overrepresentation of *DEFA3 *gene absence in the deaf group of A1555G carriers.

**Conclusion:**

Although none of the factors analysed seem to have a major contribution to the phenotype, our findings provide further evidences of the involvement of 8p23.1 region as a modifying locus for A1555G 12S rRNA gene mutation.

## Background

Mitochondrial DNA (mtDNA) mutations are an important cause of human disease and have been associated with many clinical abnormalities, including various forms of both syndromic and nonsyndromic hearing loss [1]. It has been reported that at least 5% of cases of postlingual, nonsyndromic hearing impairment are attributable to known mtDNA mutations, representing one of the most frequent causes of hearing impairment [2]. The most commonly reported nonsyndromic deafness-causing mtDNA mutations are a C insertion or deletion at position 961 [3-5], C1494T [6,7] and A1555G [8-12] in the 12S rRNA gene, and mutations A7445G [13-15], 7472insC [16,17], T7510C [18] and T7511C [4,19,20] in the tRNA^Ser(UCN) ^gene.

In particular, the A1555G mutation has been associated with aminoglycoside-induced and/or nonsyndromic hearing loss in various families of different ethnic backgrounds [8-12]. Remarkably, in Spain A1555G accounts for about 15% of all familial and sporadic cases of hearing loss, irrespective of their mode of inheritance and age of onset [21]. The phenotype associated to A1555G mutation varies considerably among matrilineal relatives, ranging from severe deafness, to moderate progressive hearing loss or even completely normal hearing. Biochemical and genetic data suggest that nuclear background may be the main factor involved in modulating the phenotypic expression of the mutation [22-24]. Extensive genome wide search revealed that nuclear modifying factors are likely to be numerous, but a region in chromosome 8p23.1 has been proposed as a putative localization for a modifier locus [22,23,25-27]. However, the gene involved has not been identified yet.

Chromosome band 8p23.1 is known to be a frequent site of chromosomal rearrangements mediated by low copy repeats (LCRs) or segmental duplications (SDs). It has been described that as many as one in four individuals from the general population carry a 4.7 Megabase (Mb) inversion of the region [28-30]. A high density of genes are present in the region, and copy number variability (CNV) involving both α-defensin (*DEFA1 *and *DEFA3*) and β-defensin (*DEFB4*, *DEFB103 *and *DEFB104*) genes has been well detected and characterized [31-34].

The objective of the present work was to analyse in detail the contribution of different 8p23.1 genetic elements to the phenotypic variability observed in deaf patients with mitochondrial 12S rRNA A1555G mutation. The analysis has focused on three different genomic features: *DEFA3 *gene absence, claudin23 (*CLDN23*) mutational analysis and the putative function of a ribosomal mitochondrial protein pseudogene (*MRPS18CP2*). These genes were selected after an exhaustive screening of the region looking for candidates as genetic modifiers of A1555G associated phenotype. Defensins were chosen because of their close proximity to the positive linkage region and *CLDN23 *and *MRPS18CP2 *were selected on the basis of their putative biological function.

## Methods

### Patients and samples

Familial cases of sensorineural hearing loss have been collected from different Spanish clinical centres with the aim to study the molecular basis of hearing loss associated to mtDNA A1555G mutation. The analysis was performed on 213 patients, from 55 pedigrees with A1555G mutation and 336 Spanish controls. The Spanish control samples were unrelated blood donor controls, all of Caucasian origin. Informed consent was obtained from all participants prior to their participation in the study, in accordance with the Institutional Review Board and Ethic Committee.

Clinical information such as the severity and age of onset of hearing impairment, the exposure to some kind of ototoxic substances, specifically aminoglycosides, and any other medical diagnoses were evaluated from at least one member of each pedigree.

### Detection of A1555G mutation

The detection of the A1555G mutation was either by PCR amplification of a 340-bp fragment (Forward 5'-GCTCAGCCTATATACCGCCATCTTCAGCAA-3' and Reverse 5'-TTTCCAGTACACTTACCATGTTACGACTTG-3'), followed by the digestion with restriction endonuclease *Hae*III, or alternatively using Pyrosequencing™ technology (PSQ96MA) (Biotage AB, Sweden). A specific SNP assay was designed for Pyrosequencing (Forward 5'-CGACATTTAACTAAAACCCCTACGC-3', Reverse 5'-GTTGGGTGCTTTGTGTTAAGCT-3' and Sequencing 5'-CACTTACCATGTTACGACT-3' primers) and sequence identification was performed automatically by the SQA software.

### *DEFA3 *determination

A PCR amplification assay followed by restriction enzyme digestion (PCR-RFLP) has been used to discriminate *DEFA1 *and *DEFA3 *gene alleles differing by a single nucleotide. A fragment of 304 bp around C3400A PSV was PCR amplified with fluorescently labelled primers (Forward 5'-TGAGAGCAAAGGAGAATGAG-3', Reverse 5'-GCAGAATGCCCAGAGTCTTC-3') and digested with *Hae*III enzyme. About 2 μl of digestion product were added to 10 μl HiDi formamide containing ROX500 marker (Applied Biosystems) and run on an ABI 3100 capillary system (Applied Biosystems). Peaks were analysed using Genemapper software (Applied Biosystems).

### Mutational screening

The genetic screening of *CLDN23 *gene and *MRPS18CP2 *pseudogene was performed by direct sequencing. The entire coding sequence of *CLDN23 *gene was PCR-amplified in two different fragments of 483 bp (Forward 5'-CCAGGAGGGAACTAGCCTAA-3' and Reverse 5'-AGCGAGGTGACCATGAGTG-3') and 679 bp (Forward 5'-GACGAGCCCAACTTCGTG-3' and Reverse 5'-AGGCAGATTTCCATCCACAC-3'). The *MRPS18CP2 *pseudogene was PCR amplified in a single fragment spanning 543 bp (Forward 5'-CTCTGTTTACAGAAGACCTGG-3', Reverse 5'-TTTTAATCTAAAATCCATGTAGCAAA-3'). The resulting PCR products were sequenced using an ABI PRISM^® ^3730 *xl *DNA Analyzer and ABI PRISM^® ^BigDye Terminator v3.1 Sequencing Kit (Applied Biosystems).

### Analysis of *MRPS18CP2 *expression

Analysis of *MRPS18CP2 *expression was assessed by RT-PCR. We used total RNA isolated from lymphoblastoid cell lines of general population subjects as well as total adult RNA from ovary, liver, spleen, lung, placenta, kidney, thymus, heart, skeletal muscle, testes, colon (Stratagene) and brain (Ambion). We employed 1 μg of total RNA for reverse transcription using SuperScript First Strand Synthesis System (Invitrogen). Reverse transcribed RNA was then PCR amplified using specific primers for *MRPS18CP2 *(Forward 5'-TGTTACAACCTTTAGGGTCCTTG-3', Reverse 5'-AGAGGTTGTTCACAATATAAAC-3').

### Statistical analysis

To compare the proportion of *DEFA3 *absence in the different groups, between groups chi-square test was performed.

Family based association tests were performed using FBAT package [35]. FBAT decomposes large pedigrees into individual nuclear families which are treated as independent in most of the calculations. The analysis was performed with 111 nuclear families, which belong to 33 large pedigrees, from which we have detailed phenotypic information and were suitable for being analysed with FBAT package. Bonferroni correction was used to account for multiple testing, correcting for the number of tests performed by the FBAT software.

For all statistical analysis performed, subjects were classified as affected or unaffected according to the available clinical data. The phenotype of subjects with reported aminoglycoside exposure was considered unknown.

The *DEFA3 *absence analysis was performed independently from the *CLDN23 *and *MRPS18CP2 *tests, considering only two different possible genotypes: present (with at least one copy of *DEFA3 *gene) or absent (without *DEFA3*). Heterozygotes for *DEFA3 *absence were only annotated in those cases were the genotype could be inferred from the pedigree data.

## Results

### α-defensin cluster is located in the positive linkage region on chromosome 8p23.1

Bykhovskaya and colleagues identified chromosome 8p23.1 as a major modifying locus for hearing loss phenotype associated to A1555G mutation [26,27]. Neither a gene nor a genetic factor involved has been found, regardless of intensive screening of the region.

**Figure 1:**
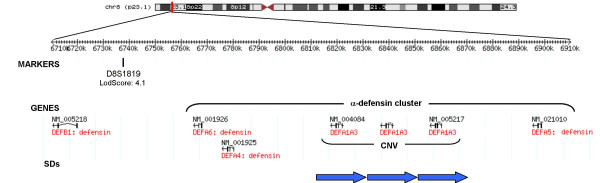
**Schematic representation of the α-defensin gene cluster on human chromosome 8p23.1**. The marker with higher lodscore in the linkage analysis is localized as well as all the genes in the region and the segmental duplication (positions are based on hg17, May 2004 genome assembly).

Genomic organization of chromosome 8p23.1 is characterized by the existence of blocks of segmental duplications flanking the region, which are known to mediate a 4.7 Mb inversion [28]. The microsatellite markers with highest lodscores in the linkage analysis [26,27] are located telomerically with respect to the inverted region and within a cluster of α-defensin genes (Figure 1).

The α-defensin cluster consists of five α-defensin genes (*DEFA6*, *DEFA4, DEFA1*, *DEFA3 *and *DEFA5*), five α-defensin pseudogenes (*DEFA8P*, *DEFA9P*, *DEFA10P*, *DEFA11P *and *DEFA7P*) and one θ-defensin pseudogene (*DEFT1P*) [36]. Three copies of a 19-kb repeat unit or copy number variant (CNV) were identified within the α-defensin cluster, which correspond to the *DEFA1A3 *CNV (based on May 2004 genome assembly). Each of the 19-kb repeats contained a copy of the *DEFA1 *or *DEFA3 *genes, but *DEFA3 *gene is known to be completely absent in a significant proportion of the population [26,27,32,34]. The description of these genomic features is relevant for the search of genetic modifying factors for A1555G mutation. Both, the presence of the polymorphic inversion and CNVs involving the α-defensin gene cluster could influence the phenotypic manifestation of deafness linked to A1555G mutation.

With the aim to investigate the role of *DEFA3 *absence in the phenotypic manifestation of A1555G mutation, we analysed the absence of *DEFA3 *gene in a group of 55 hearing impaired families or sporadic subjects with A1555G mutation (213 subjects; 135 deaf and 78 hearing) and 336 unrelated blood donor controls, all of Caucasian origin. Twenty-one of the families analysed were previously included in the whole-genome linkage analysis performed by Bykhovskaya and colleagues [26,27]. In this study, the families with non-parametric lodscore (GeneHunter) above 0.8 were considered linked to chromosome 8p23.1, and below 0 unlinked. Using these criteria, seven of the families tested (55 subjects; 31 deaf and 24 hearing) were considered linked to 8p23.1 and 14 (48 subjects; 30 deaf and 18 hearing) considered unlinked.

The frequency of individuals lacking *DEFA3 *in a control population was determined. A group of 336 subjects were tested for the absence of *DEFA3*, and found 42 individuals in whom *DEFA3 *gene was absent (12.5%). No differences were found in the rate of *DEFA3 *absence between deaf and hearing subjects in any of the situations considered: whole set of families, index cases versus control population individuals or subjects from families linked to 8p23.1 region versus controls (Table 1). The data were also analysed using a family based association test[35] under a recessive mode of inheritance, as *DEFA3 *complete absence is the only situation which could be unambiguously determined with our assay. In this case, an over-representation of *DEFA3 *absence was found in the affected group (Z = 2.36; p = 0.018) (Table 2). No distinction between linked and unlinked families was possible in this case, because of lack of statistical power to perform the calculations, as FBAT is based on the analysis of large sample groups.

**Table 1:** *DEFA3 *gene absence in A1555G patients and control subjects.

**SAMPLES**	**Phenotype**	***DEFA3***	**NO *DEFA3***	p-value*
A1555G carriers (n = 213)	Deaf (n = 135)	115 (85%)	20 (15%)	0.678
	Hearing (n = 78)	69 (88%)	9 (12%)	

A1555G carriers (n = 213)	Deaf & Hearing	184 (86%)	29 (14%)	0.283
A1555G index cases (n = 55)	Deaf	45 (82%)	10 (18%)	0.697
A1555G linked samples (n = 55)	Deaf & Hearing	52 (95%)	3 (5%)	0.171
Controls (n = 336)		294 (87.5%)	42 (12.5%)	

* Between groups chi-square p-value resulting from the comparison of deaf vs hearing carriers or carriers vs. control population subjects.

**Table 2:** Family based association study of *DEFA3 *gene absence in A1555G families.

**Marker**	**Genotype**	**Freq**	**Fam^#^**	**S**	**E(S)**	**Var(S)**	**Z**	**P**
DEFA3	present	0.689	9	8.00	6.83	2.69	0.71	0.477
	absent	0.311	9	11.00	6.67	3.36	2.36	0.018**

^# ^Number of informative families; S, observed transmission of genotype to affected offspring;

E(S), expected transmission under Mendelian inheritance; Var(S), variance; P, two-tailed P value;

**Significant P value after Bonferroni correction (P < 0.025).

### *CLDN23 *gene is not involved in the phenotypic manifestation of A1555G

Claudins are a multigene family consisting of more than 20 members. They function as cell adhesion molecules working at tight junctions. An important function in the inner ear has been postulated for several claudin genes [37,38]. Taking into account the function of other claudin family members and the fact that *CLDN23 *gene is located nearby (1.8 Mb) the defined linkage region in chromosome 8p23.1, it was selected for mutational screening as a modifier candidate gene for A1555G deafness phenotype.

**Figure 2:**
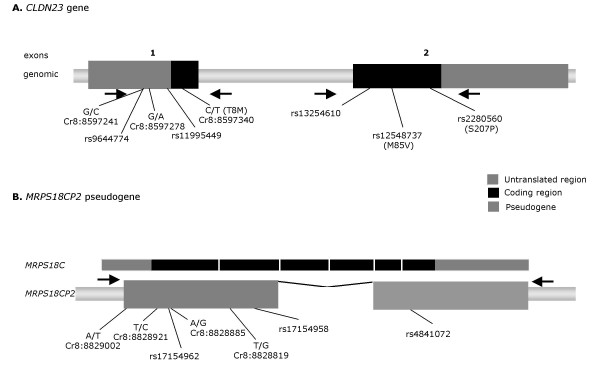
**Genetic variants identified in *CLDN23 *gene (A) and *MRPS18CP2 *pseudogene (B)**. The rs entry for the previously described SNPs or the nucleotide positions for the new identified SNPs are given. Arrows represent the position of the primers used for the PCR amplification of the corresponding genomic fragments.

Sequencing of the *CLDN23 *gene coding sequence and flanking regions in A1555G pedigrees resulted in the identification of eight sequence variants or polymorphisms, five of them already reported in public databases (Figure 2). Three of the changes resulted in an amino acid change, but none of them was identified in homozygosity, neither the variants were found to segregate with the phenotype in the pedigrees where they were identified. In addition, a deletion of 12 bp in the 5'UTR of the gene was identified in heterozygosity in one deaf sample. However, the pedigree was not informative enough to state whether it has a role in the deafness phenotype.

Although none of the variants segregated with the deafness phenotype in the analysed families, to completely rule out the involvement of *CLDN23 *gene as a modifying factor for A1555G mutation, a family based association test was performed (Table 3). The test could be only performed for two of the variants, as the others were found in a small number of samples. No significant association was found for any of the SNPs comparing the expected vs. observed transmission of each possible genotype (Table 3).

**Table 3:** Family based association study of *CLDN23 *and *MRPS18CP2 *in A1555G families.

**Gene**	**Marker**	**Genotype**	**Freq**	**Fam^#^**	**S**	**E(S)**	**Var(S)**	**Z**	**P**
*CLDN23*	rs9644774	GG	0.38	5	4.00	4.98	1.32	-0.82	0.41
		GA	0.38	10	7.00	7.40	2.93	-0.24	0.81
		AA	0.24	8	7.00	5.65	2.21	0.91	0.36
	rs11995449	GG	0.56	6	7.00	6.17	2.06	0.58	0.56
		GA	0.36	7	5.00	6.83	2.44	-1.17	0.24
		AA	0.08	2	NA				

*MRPS18CP2*	rs4841072	AA	0.38	8	13.00	8.78	3.48	2.26	0.02*
		AC	0.31	9	6.00	9.28	4.03	-1.63	0.10
		CC	0.31	3	NA				
	rs17154962	CC	0.85	5	4.00	6.28	2.00	-1.60	0.10
		CT	0.15	5	7.00	4.55	2.17	1.67	0.09
		TT	0.00	2	NA				

SNPs with less than 5 informative families were excluded from the analysis. NA; not applicable.

^# ^Number of informative families; S, observed transmission of genotype to affected offspring;

E(S), expected transmission under Mendelian inheritance; Var(S), variance; P, two-tailed P value;

*Significant p value < 0.05.

### *MRPS18C *pseudogene located on 8p23.1 is expressed in humans

**Figure 3:**
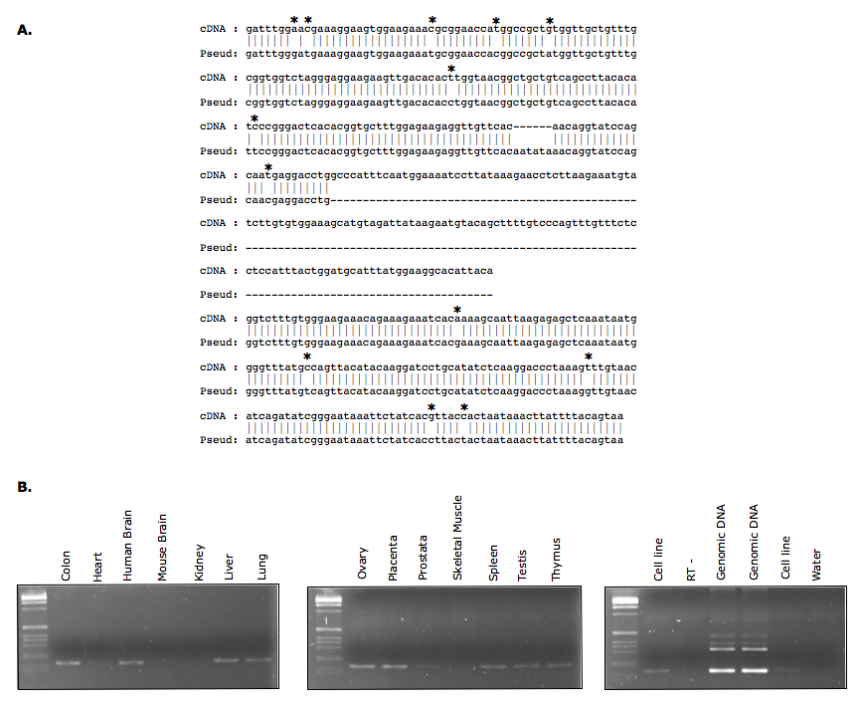
***MRPS18CP2 *sequence and expression analysis**. (A) Alignment of *MRPS18CP2 *pseudogene with *MRPS18C *mRNA (GenBank accession number NM_016067). Asterisks indicate sequence changes between the gene mRNA and the chromosome 8p23.1 pseudogene. (B) RT-PCR experiments showing expression of a transcript containing MRPS18CP2 pseudogene in different tissues.

Pseudogenes, in the case of protein-coding genes, are gene copies that have lost the ability to code for a protein. A processed pseudogene, i.e. made through mRNA retrotransposition, derived from mitochondrial ribosomal protein S18C gene (*MRPS18C*) was identified 2 Mb centromeric from *D8S1819*, the marker with a highest positive linkagee score on chromosome 8p23.1. The *MRPS18CP2 *pseudogene on chromosome 8p23.1 spans 293 bp, corresponding to the whole coding region of exons 1, 2, 5 and 6 of *MRPS18C *gene, but lacking all introns and exons 3 and 4. *MRPS18CP2 *pseudogene shares 96,9% homology with *MRPS18C *nucleotide coding sequence. There are 13 nucleotide alterations and a 6 bp deletion compared to *MRPS18C *gene (Figure 3).

Despite lacking the original promoter, a processed pseudogene can occasionally be transcribed [39]. In the public databases, neither mRNAs nor ESTs are annotated for *MRPS18CP2 *pseudogene in chromosome 8p23.1. To check whether *MRPS18CP2 *is transcribed, its expression was assessed by RT-PCR experiments using total RNA from different human tissues, human lymphoblastoid cell lines and mouse brain. A transcript containing *MRPS18CP2 *was found to be expressed in all tested tissues, except for human kidney, human skeletal muscle and mouse brain (Figure 3).

Based on the physical localization of *MRPS18CP2*, its expression pattern and the function of its corresponding coding gene, *MRPS18CP2*, was selected for a genetic screening as a candidate to be involved in the phenotypic manifestation of A1555G mutation. The mutational screening of *MRPS18CP2 *pseudogene in A1555G pedigrees resulted in the identification of seven polymorphisms, three of them already reported in public databases (Figure 2). None of the SNPs segregate with the deafness phenotype in any of the A1555G pedigrees analysed. A family based association analysis was also performed for the two informative SNPs identified (Table 3). In the case of SNP rs4841072, an overtransmission of the AA genotype (Z = 2.26; p = 0.02) was found associated to the disease, although after Bonferroni correction statistical significance was no longer supported (Table 3).

## Discussion

Large-scale chromosomal rearrangements, such as duplications, deletions and inversions, are now known to be common in the human genome [40]. The substrates for these common rearrangements are generally highly homologous sequences, known as segmental duplications or LCRs, which flank the rearranged genomic segment [41]. To take into account genomic structural variation is crucial in linkage studies of human diseases for different reasons. First, when a fixed marker order is assumed for all individuals in an inverted region, one tends to see spurious recombination events among inversion carriers and/or to find genotyping contradictions, which may lead to discard some observations. In addition, the polymorphic genomic structure of the rearranged regions, which apart from large-scale genomic rearrangements can include sequences that vary in copy number, might complicate the mapping of putative disease genes. Chromosome 8p23.1 is such a region where a common neutral inversion mediated by clusters of olfactory-receptor genes, is present in a variable proportion of subjects, depending on the population [28-30]. The position of a major nuclear modifier gene for the deafness phenotype linked to A1555G mtDNA mutation has been localized to chromosome 8p23.1 [27], but the identification of this gene has remained elusive. This lack of progress may be partially explained because of 8p23.1 genomic organization.

In an attempt to further study the putative genetic modifying factors for A1555G mutation, including those derived from the presence of segmental duplications, we have performed a detailed analysis of three 8p23.1 candidate genetic features: *CLDN23 *gene, *MRPS18CP2 *pseudogene and *DEFA3 *gene absence. *CLDN23 *gene and *MRPS18CP2 *pseudogene were selected based on their putative biological role in the inner ear, whereas *DEFA3 *gene absence was tested due to its close location to the marker with a higher lodscore.

Claudins are essential components of tight junctions [42] and therefore, they play important roles in the physiological function of the inner ear. Tight junctions are well developed in the epithelial cell layers that delineate the inner ear compartments containing perilymph and endolymph, to prevent intercellular leakage of solutes and ions [43]. In fact, mutation of the *Claudin-14 *gene was reported to cause human hereditary deafness [38] and *Claudin-11 *null mice exhibit severe deafness associated with low endocochlear potential [37]. In addition, at least 10 species of claudins are expressed in the inner ear [44].

Pseudogenes are non-functional sequences of genomic DNA originally derived from functional genes [45]. The human genome encodes at least 79 mitochondrial ribosomal proteins from which more than 100 pseudogenes have been identified [46]. Located on chromosome 8p23.1, there is *MRPS18CP2*, a processed pseudogene of mitochondrial ribosomal protein S18C (MRPS18C). Five other pseudogenes derived from *MRPS18C *gene are located in the human genome on chromosomes 3q26.1, 8p21.3, 12p13.31, 15q11.2 and 22q13.31 respectively [46]. Interestingly, the *MRPS18C *pseudogene on chromosome 15q11.2 is located only 1-Mb apart from a microsatellite marker, which gave a positive linkage score in the analysis performed by Bykovskaya and colleagues [26]. It has been postulated that pseudogenes may play regulatory roles for the genes from which they have been derived, such as serving as a source of antisense RNA [45]. Taking all these evidences into account and regardless that the functional role of pseudogenes is not clear, *MRPS18CP2 *was considered a good candidate.

None of the identified SNPs in either *CLDN23 *or *MRPS18CP2 *segregate with the phenotype in A1555G families, but as modifying factors are likely to be multiple [25,26], this observation did not provide enough evidence to completely discard their contribution in the A1555G deafness phenotype. Thus, a family-based association test was used to analyse the genotype data from *CLDN23 *gene and *MRPS18CP2 *pseudogene. Family-based association designs are particularly attractive, since they test for linkage as well as association, avoid spurious associations caused by admixture of populations, and are convenient for investigators interested in refining linkage findings in family samples [35]. With this approach, a weak positive association with a single SNP in *MRPS18CP2 *pseudogene was found. Although most of the analysed samples come from the same geographic area, founder effects do not account for the association found as it was previously reported [47,48].

These results, although have to be taken with caution, are of great interest as they may suggest a possible role for *MRPS18CP2 *pseudogene. Three sequence variants have been found for MRPS18 protein of the small mitochondrial ribosome subunit. In analogy to bacterial ribosomes, it is likely that each mitochondrial ribosome contains a single copy of MRPS18. Therefore, the presence of three different isoforms suggests that there is a heterogeneous population of mitochondrial ribosomes, which may have different decoding properties and may be subjected to a precise regulation of its expression [47]. The existence of MRPS18 pseudogenes could play a role in the regulation of each isoform expression, for example by blocking the expression of the corresponding gene. If this is demonstrated, it could explain the tissue specificity of A1555G homoplasmic mtDNA mutation, leading to a clinical phenotype confined in the cochlea. Thus, additional studies involving typing of additional SNPs in gene-coding and regulatory regions in additional A1555G families are needed, especially in the case of pseudogenes, whose putative biological function is still unclear.

CNVs have been proposed to have an important role in the pathological variation in the human population [49]. The *DEFA1A3 *CNV is located within the region previously described to contain a major modifying locus for mutation A1555G [32,34]. On the premise that the presence of a gene in multiple copies could have a dosage effect and therefore, contribute to genetic basis of some complex disorders, it is feasible that the copy number polymorphism of α-defensin cluster could be involved in the pathogenesis associated to the A1555G mutation. An overrepresentation of *DEFA3 *gene absence was found in deaf A1555G carriers. Defensins are small cationic peptides that form an important part of the innate immune system. It is difficult to establish a direct relationship between defensin function and A1555G deaf phenotype. However, as the distinction between *DEFA1 *and *DEFA3 *is based on the typing of a single SNP (C3400A), the differences in the rate of *DEFA3 *gene absence observed between deaf and hearing carriers of A1555G mutation could be considered as a positive association signal that confirms the localization of a modifier factor.

## Conclusion

Both positive results found in *MRPS18CP2 *pseudogene and *DEFA3 *gene absence within the deaf group of A1555G carriers are weak associations, which do not demonstrate a role in the phenotype linked to A1555G mtDNA mutation. However, they provide further evidences of the involvement of 8p23.1 region as a modifying factor for A1555G mutation. Further analyses in additional families, as well as functional studies, which should shed light on the function of these genetic features, are needed in order to confirm or discard the associations found between 8p23.1 genes and A1555G hearing impairment.

## Competing interests

The author(s) declare that they have no competing interests.

## Authors' contributions

EB carried out the molecular genetic studies, participated in the statistical analysis and drafted the manuscript. JMM carried out the statistical analysis of the data and participated in the interpretation of the data. NFG participated in the design of the study and coordination. XE conceived the study and participated in its design and coordination and helped to draft the manuscript. All authors have read and approved the final version of the manuscript.

## Pre-publication history

The pre-publication history for this paper can be accessed here:


